# 609. Implementation of Bayesian Software for Vancomycin Dosing Integrated into an Electronic Medical Record

**DOI:** 10.1093/ofid/ofab466.807

**Published:** 2021-12-04

**Authors:** Nicholas A Jantrakul, Molly M Miller, Bryan Alexander, Trevor C Van Schooneveld, Trevor C Van Schooneveld, Scott J Bergman, Scott J Bergman

**Affiliations:** 1 Creighton University/Nebraska Medicine, Omaha, NE; 2 Nebraska Medicine, Bellevue, NE; 3 University of Nebraska Medical Center, Omaha, NE

## Abstract

**Background:**

New vancomycin dosing guidelines recommend using a 24-hour area under the curve (AUC) goal of 400-600mg*h/L rather than a serum trough concentration 10-20 mg/L. This target has the potential to lower troughs and the occurrence of acute kidney injury (AKI). In summer 2020, we were the first to integrate DoseMeRx^®^ Bayesian software into Epic OneChart^®^, our electronic medical record, to assist with dosing vancomycin. We sought to determine how doses and trough concentrations changed after software implementation. The study also aimed to establish the sample size needed to assess AKI rates.

**Methods:**

This quasi-experimental study evaluated patients receiving ≥3 vancomycin doses before software integration (Q1 2020) and after (Q4 2020). Fifty patients in whom an adult 1-compartment model could be used and a trough concentration was measured were randomly selected from each period. Patients age ≤18 years, ≥200kg, or with pre-existing renal failure were excluded. AKI was defined using AKIN criteria of an increase in Scr of ≥ 0.3 mg/dL over 48 hours. Student’s t-test was used for continuous variables and Fischer exact test for categorical outcomes.

**Results:**

In Q1, 299 patients had ≥3 vancomycin doses with 107 reviewed to reach 50 meeting inclusion criteria. In Q4, 346 had ≥3 doses and 94 were reviewed to include 50 patients. The primary reason for exclusion was no trough concentration. Demographics and indications for vancomycin were well matched. Skin/soft tissue infection was the most common indication for vancomycin (n=16, both Q1 and Q4). Sixteen patients were in the ICU with 8 on vasopressors before integration. There were 10 patients in the ICU and 6 on vasopressors after. Results in Table 1 were not significantly different (all *p* >0.05). AKI occurred in 5 (10%) patients before integration and 3 (6%) after. Based on this, a sample of 1442 patients, or approximately 15 months of vancomycin AUC-based dosing, will need to be analyzed to achieve 80% power to detect a significant difference in AKI.

Table 1. Results related to vancomycin dosing before and after software integration

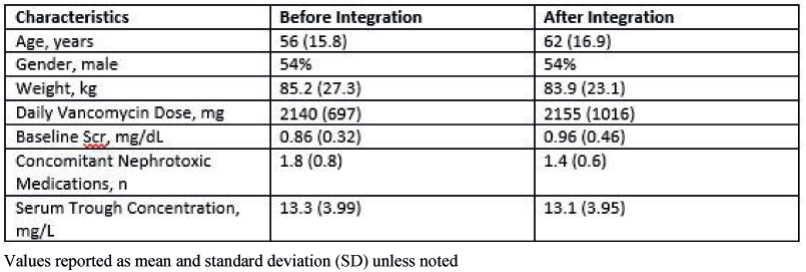

Values reported as mean and standard deviation (SD) unless noted

**Conclusion:**

Vancomycin doses and trough concentrations were similar in the two groups and AKI rates were numerically lower. This serves as an exploratory analysis to inform a larger study on the effects of integrating Bayesian dosing software for vancomycin dosing.

**Disclosures:**

**Bryan Alexander, PharmD**, **Astellas Pharma** (Advisor or Review Panel member) **Trevor C. Van Schooneveld, MD, FACP**, BioFire (Individual(s) Involved: Self): Consultant, Scientific Research Study Investigator; Insmed (Individual(s) Involved: Self): Scientific Research Study Investigator; Merck (Individual(s) Involved: Self): Scientific Research Study Investigator; Rebiotix (Individual(s) Involved: Self): Scientific Research Study Investigator **Scott J. Bergman, PharmD, FCCP, FIDSA, BCPS, BCIDP**, **Merck & Co., Inc** (Grant/Research Support) **Scott J. Bergman, PharmD, FCCP, FIDSA, BCPS, BCIDP**, Merck & Co., Inc (Individual(s) Involved: Self): Research Grant or Support

